# Unlocking Novel Ultralow-Frequency Band Gap: Assembled Cellular Metabarrier for Broadband Wave Isolation

**DOI:** 10.3390/ma15238326

**Published:** 2022-11-23

**Authors:** Xiao Liang, Fang Zhang, Jinhui Jiang, Cang He, Hongji Yang

**Affiliations:** 1 State Key Laboratory of Mechanics and Control of Mechanical Structures, Nanjing University of Aeronautics and Astronautics, Nanjing 210016, China; 2 Laboratory of Aerospace Entry, Descent and Landing Technology, CASC, Beijing 100094, China

**Keywords:** negative effective parameters, elastic metamaterial, gradient design, wave isolation, ultralow frequency

## Abstract

Admittedly, the design requirements of compactness, low frequency, and broadband seem to constitute an impossible trinity, hindering the further development of elastic metamaterials (EMMs) in wave shielding engineering. To break through these constraints, we propose theoretical combinations of effective parameters for wave isolation based on the propagation properties of Lamb waves in the EMM layer. Accordingly, we design compact EMMs with a novel ultralow-frequency bandgap, and the role of auxeticity in the dissociation between the dipole mode and the toroidal dipole mode is clearly revealed. Finally, under the guidance of the improved gradient design, we integrate multiple bandgaps to assemble metamaterial barriers (MMBs) for broadband wave isolation. In particular, the original configuration is further optimized and its ultralow-frequency and broadband performance are proven by transmission tests. It is foreseeable that our work will provide a meaningful reference for the application of the new EMMs in disaster prevention and protection engineering.

## 1. Introduction

The first two decades of the 21st century have witnessed frequent climatic anomalies and natural disasters, especially super earthquakes and their triggering of secondary disasters such as tsunamis with horrific destructive power, w hich have been a nightmare for mankind [1]. In addition, the wave energy of various types of traffic systems, large machinery, and even explosive weapons can spread through the ground and strata, and these vibrations can bring about detrimental effects on sensitive targets in specific frequency ranges. Therefore, the construction of tailored and wide-domain effective wave protection engineering has been of considerable academic and practical value.

In recent years, the effectiveness of subwavelength-scale elastic metamaterials (EMMs) for wave attenuation at the ground surface has been demonstrated in engineered experiments [2,3]. Distinguished from traditionally civil-reinforced structures [4,5,6,7], EMMs offer a new promising solution for wave protection engineering based on refraction, reflection, and artificial modulation of elastic waves [8]. More impressively, driven by burgeoning new needs, EMMs are flourishing with their unique physical properties in areas such as programmable robotics [9], lensing and super-resolution imaging [10,11,12,13], stealth [14,15,16,17,18,19], non-reciprocal wave control [20,21], and topological insulators [22,23,24,25,26,27,28]. Undoubtedly, new discoveries in the field of metamaterial physics are inspiring people to design even more magical metamaterial barriers (MMBs) to accomplish better seismic resistance or vibration isolation. In terms of application, the most pivotal property for EMMs is the presence of bandgaps. According to the formation mechanism of the bandgaps, it can be physically classified into local resonance (LR) and Bragg scattering (BS) mechanisms. In general, all the above band gaps are the result of a combination of Mie scattering and structural periodicity, but the former is oriented to scatterer resonance while the latter is more dependent on periodicity [29,30,31,32,33,34]. Of course, the resonance of monopole, dipole, and even quadrupole features introduced by the multiple inclusions also adds diversity to the band structure.

Most of the time, in the semi-infinite space model, it is believed that the main component of the destructive energy is carried by surface waves, so a great deal of research has focused on the use of buried mass resonators or surface resonators to attenuate Rayleigh waves [35,36,37,38]. What is less concerned is that, in fact, in the natural multi-layered geological structures, it has been proved that the wave energy can propagate in the Lamb mode in the harder stratum [39]. Moreover, in civil engineering, there are often cases where stratified strata are constructed and, in addition to above-ground buildings, underground structures such as storage containers also need to be protected, so the isolation of Lamb waves is also worth studying [40,41,42,43]. Although a great deal of experience has been gained with surface wave isolation engineering, designing compact MMBs with ultralow frequency (especially 0.1–20 Hz) and broadband Lamb wave attenuation is still challenging.

In terms of low-frequency and even ultralow-frequency vibration isolation of seismic waves, researchers have explored this in depth from multiple perspectives. For example, wide zero-frequency stop bands have been observed in a deep medium due to the strong confinement between the pile and the soil substratum, which offered the possibility of ultralow-frequency seismic wave modulation [44]. Later, in Matryoshka-like structures with different numbers of tube layers, the generation of new bandgaps accompanied by frequency shifts to lower-frequency ranges was found [45]. In particular, Ungureanu et al. [46] achieved seismic wave modulation in the frequency range from 1 Hz to 40 Hz using auxetic-like metamaterials via investigating the bandgap properties of auxetic materials. Further, a square cross-sectional EMM composed of auxetic foam and steel was recently proposed [47] and its ability to attenuate low-frequency Lamb waves was verified by simulations on a plate model, although its attenuation range was not wide. Noteworthy research advances have also shown that the intercalation of novel materials such as auxetic materials into metamaterial structures with different topological configurations can lead to designable and extraordinary mechanical properties [48,49,50,51,52,53,54,55]. The above studies provide ideas for solving the challenge of restraining seismic waves at low and even ultralow frequencies. Certainly, the low-frequency bandgap caused by inertial amplification can also be used to isolate seismic waves, but the additional mechanical structure introduced may greatly increase its cost [56,57].

When considering the optimization of attenuation range, the gradient effect [58,59] and the rainbow trapping [60,61] have been proposed and validated in order to achieve a wider range of wave attenuation or vibration energy absorption. It is also noteworthy that Muhammad and Lim et al. [62,63,64] introduced embedded or semi-embedded multi-resonators, which made an enlightening contribution to the realization of broadband seismic Rayleigh wave attenuation. Furthermore, the research on the EMMs composed of square steel profiles illustrates that it can generate a relatively wide complete bandgap, but the structural size of this scheme is still too large to be used in practice [65]. Actually, the wave attenuation bandwidth caused by the local resonant bandgap alone, especially the first bandgap (FBG), is relatively narrow [66,67,68]. Therefore, researchers have begun to pay attention to the second bandgap (SBG) in the higher-frequency band or the relatively wide bandgap formed by the BS mechanism [69,70,71]. If the advantages of both can be exploited, it will be possible to contrive MMBs with ultralow-frequency and ultra-broadband wave attenuation capabilities. Recently, experimental results have shown that coupling FBG and SBG can create wider Rayleigh wave attenuation, where the distance between the different bandgaps is critical [72]. In the latest research on the introduction of strong nonlinearity into resonators arranged on free surfaces, the dispersion of low- and higher-order harmonics spreading through the metasurface has been found, which provides an opportunity to broaden the bandgap, but it is still difficult to reduce the effective frequency to an ultralow level [73,74].

**Figure 1:**
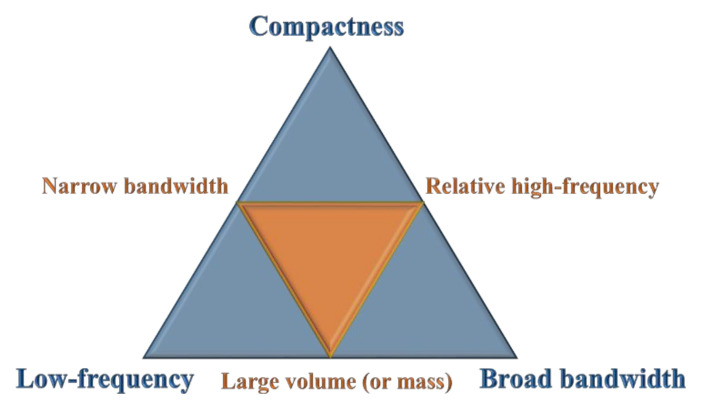
Diagram of the impossible trinity in MMB design.

At present, to some extent, the compactness of MMBs (or artificial embedded structures), the low operating frequency, and the broadband wave attenuation capability seem to constitute the impossible trinity (as shown in Figure 1) that shackles the further application of EMMs in vibration prevention engineering. In other words, in traditional designs, the true situation is more likely to occur on three sides of a larger triangle (i.e., the sides where the vertices of the orange triangle are located). That is, the three main properties are often satisfied with at most two of them.

To break through these constraints, this work aims to design a more compact EMM to isolate Lamb waves, and furthermore, reveal and utilize the new ultralow-frequency bandgap without introducing additional mass or complex devices. Here, the emergence of the ultralow-frequency bandgap is induced by the negative Poisson’s ratio of the substrate material, inspired by the dissolution of degeneracy near the Dirac point that opens in graphene-like two-dimensional materials. In Section 2, we investigate the propagation behavior of Lamb waves in the EMM layer and indicate effective parameter combinations to isolate Lamb waves. Then, three EMM cells with different scatterer cross-section shapes are proposed according to the effective parameters. In Section 3, we discuss in detail the influence of factors such as geometry and material properties on the band structure and explain them based on the effective parameter theory. Further, in Section 4, we construct the assembled MMB based on an improved gradient design and verify its ultralow-frequency wave isolation performance by transmission analysis. Finally, the conclusions of this work are set out in Section 5.

## 2. Methods and Models

### 2.1. Propagation Behavior of Lamb Waves in EMM Layer

**Figure 2:**
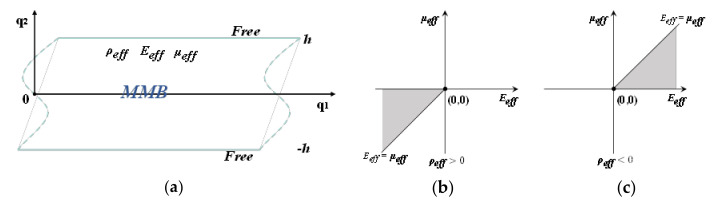
(**a**) Schematic diagram of the EMM layer; (**b**) quadrant diagram of the effective parameter combinations when the effective mass density is positive or (**c**) negative.

Figure 2a illustrates a schematic diagram of the EMM layer that extends infinitely in the q_1_ direction and takes up a thickness of 2*h* in the q_2_ direction, while its upper and lower surfaces are non-traction boundaries. In addition, *ρ_eff_*, *E_eff_*, and *μ_eff_* denote the effective mass density, effective P wave modulus, and effective S wave modulus of the medium, respectively. When the Lamb wave propagates within the layer shown in Figure 2a, the preliminary displacement potential functions can be written as [75]

(1) $$\frac{\partial^{2}\varphi }{\partial q_{1}^{2}}+\frac{\partial^{2}\varphi }{\partial q_{2}^{2}}=\frac{1}{c_{L}^{2}}\frac{\partial^{2}\varphi }{\partial t^{2}}$$

(2) $$\frac{\partial^{2}\psi }{\partial q_{1}^{2}}+\frac{\partial^{2}\psi }{\partial q_{2}^{2}}=\frac{1}{c_{T}^{2}}\frac{\partial^{2}\psi }{\partial t^{2}}$$

where $c_{L}=\sqrt{E_{eff}/\rho_{eff}}$ and $c_{T}=\sqrt{\mu_{eff}/\rho_{eff}}$ are the corresponding effective wave velocities. Moreover, the displacement potentials can be expressed as

(3) $$\varphi =[A_{1}\sin(k_{L}q_{2})+A_{2}\cos(k_{L}q_{2})]e^{j(k_{1}q_{1}-\omega t)}$$

(4) $$\psi =[B_{1}\sin(k_{T}q_{2})+B_{2}\cos(k_{T}q_{2})]e^{j(k_{1}q_{1}-\omega t)}$$

where $k_{L}=\sqrt{\omega^{2}/c_{L}^{2}-k_{1}^{2}}$ represents the wave numbers of the P wave in the thickness direction, and $k_{T}=\sqrt{\omega^{2}/c_{T}^{2}-k_{1}^{2}}$ denotes S wave numbers in the same direction (i.e., the q_2_ direction). In addition, *ω* is the circular frequency and *k*_1_ is the wave number in the q_1_ direction.

It should be noted that Equations (3) and (4) contain both symmetric and antisymmetric fluctuation solution components. Based on the stress conditions at the free boundaries (q_2_ = ±*h*), the classical Rayleigh-Lamb equation can be obtained as

(5) $$\frac{\tan(k_{T}h)}{\tan(k_{L}h)}=-\frac{4k_{1}^{2}k_{L}k_{T}}{{\left(k_{T}^{2}-k_{1}^{2}\right)}^{2}}$$

(6) $$\frac{\tan(k_{T}h)}{\tan(k_{L}h)}=-\frac{{\left(k_{T}^{2}-k_{1}^{2}\right)}^{2}}{4k_{1}^{2}k_{L}k_{T}}$$

Among them, the wave number *k*_1_ will possess a finite real or pure imaginary form at a given frequency, but there are infinite solutions in the complex field. Then, considering the phase velocity and the frequency-thickness product, Equations (5) and (6) are normalized to obtain the dispersion relations of symmetric and antisymmetric Lamb waves, as follows:

(7) $$\frac{\left(1-e^{\frac{\Omega }{c}\sqrt{1-\frac{c^{2}}{c_{L}^{2}}}})\cdot (1+e^{\frac{\Omega }{c}\sqrt{1-\frac{c^{2}}{c_{T}^{2}}}}\right)}{\left(1+e^{\frac{\Omega }{c}\sqrt{1-\frac{c^{2}}{c_{L}^{2}}}})\cdot (1-e^{\frac{\Omega }{c}\sqrt{1-\frac{c^{2}}{c_{T}^{2}}}}\right)}=\frac{4\sqrt{\left(1-\frac{c^{2}}{c_{L}^{2}})\cdot (1-\frac{c^{2}}{c_{T}^{2}}\right)}}{{\left(\frac{c^{2}}{c_{T}^{2}}-2\right)}^{2}}$$

(8) $$\frac{\left(1-e^{\frac{\Omega }{c}\sqrt{1-\frac{c^{2}}{c_{L}^{2}}}})\cdot (1+e^{\frac{\Omega }{c}\sqrt{1-\frac{c^{2}}{c_{T}^{2}}}}\right)}{\left(1+e^{\frac{\Omega }{c}\sqrt{1-\frac{c^{2}}{c_{L}^{2}}}})\cdot (1-e^{\frac{\Omega }{c}\sqrt{1-\frac{c^{2}}{c_{T}^{2}}}}\right)}=\frac{{\left(\frac{c^{2}}{c_{T}^{2}}-2\right)}^{2}}{4\sqrt{\left(1-\frac{c^{2}}{c_{L}^{2}})\cdot (1-\frac{c^{2}}{c_{T}^{2}}\right)}}$$

Theoretically, when the wave number *k*_1_ is a positive real number, which implies a travelling wave mode that can propagate efficiently along the direction of the elastic layer (i.e., the q_1_ direction), then the wave number *k_L_* (or *k_T_*) of the P wave (or S wave) in the thickness direction can only be a positive real or pure imaginary number.

As there are many combinations between the positive and negative values of the three effective parameters of the EMM layer, the traveling wave modes determined by Equations (7) and (8) are also various. In general, due to the frequency dependence of the effective medium parameters, we often design the imaginary phase velocity to convert the original traveling wave into the evanescent mode. Here, we can list all parameter combinations with imaginary effective wave velocities, as shown in Table 1.

However, due to the boundary effect at the free boundary of the elastic medium, the coupling of the P wave and SV wave will be induced, and the P or SV wave potential in the thickness direction can exhibit a surface traveling wave mode in the form of an evanescent wave [76]. In other words, even in the EMM layer with double imaginary wave velocities, where both *k_L_* and *k_T_* are imaginary, traveling wave transmission can still occur. Hence, further determination of the range of effective parameters is needed to inform the design of metamaterials with stable Lamb wave isolation. Considering the case where both wave velocities are imaginary ($c_{L}^{2}<0\&c_{T}^{2}<0$), it can be determined that the effective modulus ratio is $\sigma =E_{eff}/\mu_{eff}=c_{L}^{2}/c_{T}^{2}>0$. Meanwhile, after introducing the simplified parameters $\alpha =\sqrt{1-c^{2}/c_{L}^{2}}$, $\beta =\sqrt{1-c^{2}/c_{T}^{2}}$, and γ=Ω/c, Equations (7) and (8) can be refined to

(9) $$\frac{\left(e^{\gamma \alpha }-1)\cdot (e^{\gamma \beta }+1\right)}{\left(e^{\gamma \alpha }+1)\cdot (e^{\gamma \beta }-1\right)}=\frac{4\alpha \beta }{{\left(\beta^{2}+1\right)}^{2}}$$

(10) $$\frac{\left(e^{\gamma \alpha }-1)\cdot (e^{\gamma \beta }+1\right)}{\left(e^{\gamma \alpha }+1)\cdot (e^{\gamma \beta }-1\right)}=\frac{{\left(\beta^{2}+1\right)}^{2}}{4\alpha \beta }$$

Clearly, if the waveguide exists, then *c* > 0 and, hence, *α* > 1, *β* > 1, and *γ* > 0. Next, when *σ* ≥ 1, there is 1 < *α* ≤ *β*. To analyze the existence of the solution, the following function is constructed with Equation (9) as an example:

(11) $$f(\alpha )=\frac{(e^{\gamma \alpha }-1)\cdot (e^{\gamma \beta }+1)\cdot {\left(\beta^{2}+1\right)}^{2}}{\left(e^{\gamma \alpha }+1)\cdot (e^{\gamma \beta }-1)\cdot (4\alpha \beta \right)}-1$$

where *f* = 0 is equivalent to Equation (9). Then, the derivative of the function to α can be expressed by

(12) $$\frac{df}{d\alpha }=\frac{e^{\gamma \beta }+1}{e^{\gamma \beta }-1}\cdot \frac{{\left(\beta^{2}+1\right)}^{2}}{4\beta }\cdot \frac{e^{\gamma \alpha }(2\gamma \alpha -e^{\gamma \alpha })+1}{\alpha^{2}{\left(e^{\gamma \alpha }+1\right)}^{2}}$$

where γα>0, so $e^{\gamma \alpha }(2\gamma \alpha -e^{\gamma \alpha })+1<0$, and then we have df/dα<0.

In addition, when we assume that α=β, $f\left(\beta \right)={\left(\beta^{2}-1\right)}^{2}/4\beta^{2}>0$ holds. Hence, in the case of 1 < *α* ≤ *β*, we obtain the following relationship:

(13) f(α)≥f(β)>0

Therefore, *f* = 0 is impossible, which implies that Equation (9) has no solution when *σ* ≥ 1, that is, there is no corresponding traveling wave mode at this time. Likewise, we can use the construction function method to derive that the solution of Equation (10) is also nonexistent under the case of *σ* ≥ 1. Further, for the case of 0 < *σ* < 1, the solutions of Equations (9) and (10) can be found based on similar function processing or numerical methods. Thus, as shown in Figure 2b,c, these two specific combinations of the effective parameters $\rho_{eff}>0,E_{eff}<0,\mu_{eff}<0$ and $E_{eff}/\mu_{eff}\geq 1$, and $\rho_{eff}<0,E_{eff}>0,\mu_{eff}>0$ and $E_{eff}/\mu_{eff}\geq 1$, can be used to tailor EMMs to efficiently isolate Lamb waves.

### 2.2. MMB Design and Band Structure Calculation

**Figure 3:**
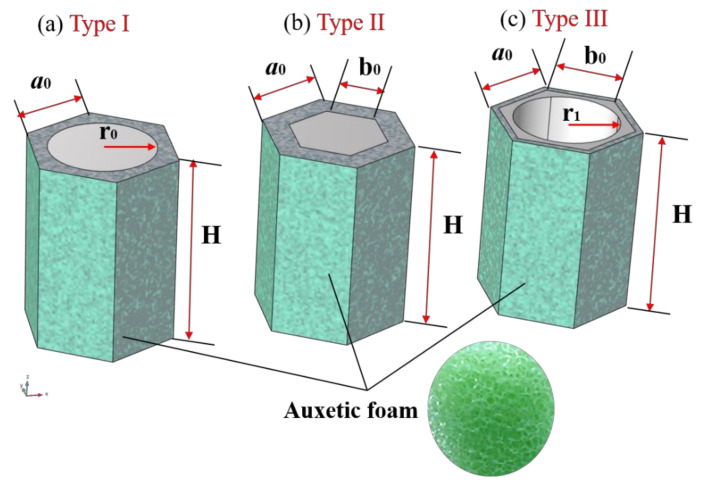
Schematic diagram of the metamaterial units with: (**a**) solid cylindrical scatterers (Type I), (**b**) solid hexagonal cross-sectional scatterers (Type II), and (**c**) hexagonal cross-sectional scatterers containing hollow circular holes (Type III).

Three types of metamaterial units with hexagonal cross-sections proposed can be seen as composites of substrate-wrapped scatterers, and their structural characteristics are drawn in Figure 3. In more detail, the dimensional parameters and material components of each unit are marked in Figure 3. Among them, the green filled area represents the substrate, which is composed of auxetic foam, while the light-gray-filled area is the scatterer, and its material is steel. Specifically, the dimensional parameters of the units are listed in Table 2, while the parameters of the materials can be found in Table 3. In addition, it should be noted that the feature size (*a*_0_) of these units is 2 m, while the other size parameters vary within a certain range as required.

**Figure 4:**
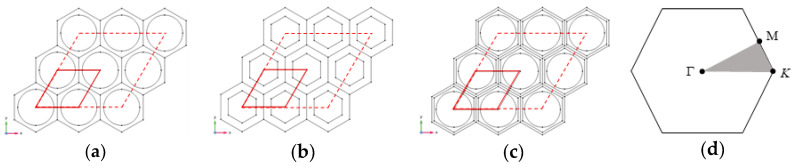
2D view of X-Y plane of the periodic MMB composed of: (**a**) unit cell of Type I, (**b**) unit cell of Type II, and (**c**) unit cell of Type III; (**d**) the first Brillouin zone with the irreducible part (light grey triangle of vertices K-Γ-M-K).

Here, we consider the propagation of Lamb waves in the plane and carry out numerical analysis by means of the FEM. As shown in Figure 4a–c, as the MMB composed of the above-mentioned metamaterial units can be regarded as the periodic system in the two-dimensional plane, the dispersion of the unit cell with the lattice constant *a*_0_ can be studied by applying the periodic boundary condition (PBC). The periodic boundary is marked by solid red lines; in other words, the diamond-shaped region represents the unit cell while the red dotted line encircles the supercell. The governing equation of elastic wave propagation in the plane is given by [77]

(14) $$\nabla \cdot \left[C\left(r\right):\nabla u\right]+\rho \left(r\right)\omega^{2}u=0$$

where *r*, *u*, C(*r*), and *ρ*(*r*) represent the position vector, the displacement vector, the elastic tensor, and mass density, respectively; ω is circular frequency; “⋅”and “:” denote the vector dot product and double-dot product, respectively; ∇ is the differential operator.

Referring to the Floquet-Bloch theory to research the dynamic behavior of waves in periodic unit cells, the displacement vector in Equation (14) can be expressed in the form [78]:

(15) $$u\left(r\right)=e^{i\left(k\cdot r\right)}u_{k}\left(r\right)$$

where *k* is the Floquet-Bloch wave vector in the first Brillouin zone of the reciprocal lattice, and *i* is the imaginary unit. Specifically, *u_k_*(*r*) represents the periodic modulation function of the displacement vector defined as

(16) $$u_{k}\left(r\right)=u_{k}\left(r+a\right)$$

where *a* is the lattice translation vector. Substituting Equation (16) into Equation (15), the periodic boundary condition for the unit cell is obtained as

(17) $$u\left(r+a\right)=e^{i\left(k\cdot a\right)}u\left(r\right)$$

Associating Equations (14) and (17), the dispersion relation of the periodic system can be transferred into an eigenvalue equation:

(18) $$\left(S\left(k\right)-\omega^{2}M\left(k\right)\right)U=0$$

where S(*k*) and M(*k*) are the stiffness and mass matrix of the unit cell, respectively.

Then, the dispersion relation can be regarded as the implicit function between the wave vector and the eigenfrequency. Obviously, when calculating all wave propagation modes, the wave vector k should sweep the boundary of the first irreducible Brillouin zone (IBZ), as shown in Figure 4d. It should be noted that when calculating the dispersion characteristics of supercells, the sweep range of the wave vector will no longer be limited in the IBZ. Whether to take unit cell or supercell for calculation depends on the specific modal observation requirements. In this study, the frequency domain solver of finite element software is used to analyze the dispersion relationships.

## 3. Parametric Analysis and Discussion

Before evaluating the shielding performance of the MMB against Lamb waves, the band structures of the various metamaterial units are studied to make comparative analysis and optimize the design. Specifically, the cross-sectional shape and the hollowness ratio of the scatterer, the filling ratio, and materials parameters of the units are discussed in detail in this section.

### 3.1. Topological Configuration of the EMMs

**Figure 5:**
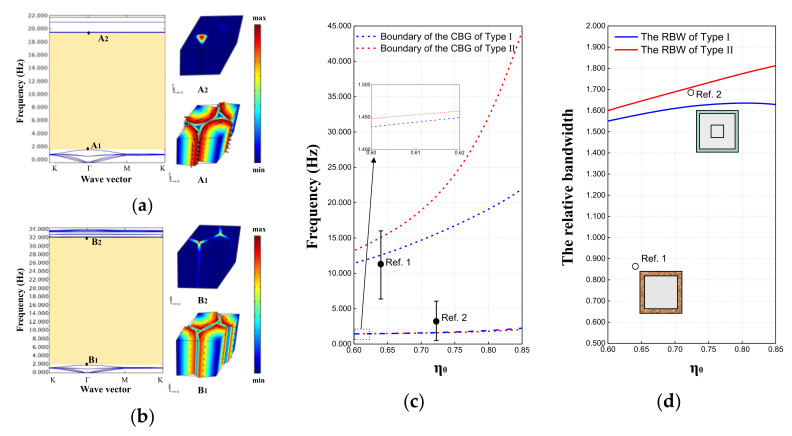
Dispersion relations and concerned vibration mode shapes of EMMs in (**a**) Type I and (**b**) Type II when η_0_ = 0.81 and H = 2.5*a*_0_; (**c**) the bandgap boundaries and (**d**) the RBW of Type I and Type II EMM when the filling ratio varies between 0.60 and 0.85.

Fundamentally, the microstructure and arrangement of metamaterial units together determine the topological characteristics of the MMB system. In this section, the dispersion curves of metamaterials composed of type I and type II units are analyzed to explore the influence of the cross-sectional shape of the scatterer on the bandgap borders and relative band width (RBW). More specifically, we define RBW as $2\left(f_{u}-f_{l}\right)/\left(f_{u}+f_{l}\right)$, where $f_{u}$ and $f_{l}$ are the upper and lower boundary frequencies of the bandgap, respectively. It should be added that for these two types of metamaterial units, the filling ratio here is defined as the volume fraction of the scatterer in the unit body. Further, the filling ratio (η_0_) of the unit can be expressed as $\eta_{0}=V_{\mathrm{scatterer}}/V_{\mathrm{unit}}$. In other words, the filling ratio is proportional to $(r_{0}/a_{0}{)}^{2}$ and $(b_{0}/a_{0}{)}^{2}$ in unit bodies (type I and II) of the same height, respectively. In view of the principle of controlling variables, the default unit height H is set to 2.5*a_0_*. In addition, in Figure 5, the final results of Zhang et al. [65] are denoted as Ref. 1, and the optimal results of Huang et al. [47] are labeled as Ref. 2 in order to facilitate the comparison of the computational results in this paper with those of previous studies.

With the calculation method introduced in Section 2, typical dispersion curves of the metamaterials composed of type I and type II units are presented in Figure 5a,b, respectively. At this time, the filling ratio η_0_ is set as 0.81. It can be clearly seen from Figure 5a,b that the first complete bandgap (CBG) obtained by the two types of metamaterials lies between 1.920 and 19.402 Hz, and 1.794 and 31.613 Hz, respectively. Meanwhile, the vibration modes corresponding to the bandgap boundaries are depicted on the right side of the dispersion diagram. For the convenience of observation, we synthesize the displacement in the vibration mode diagram and mark it with black arrows. It is not difficult to find from A1 and B1 mode diagrams that, corresponding to the lower boundary of the bandgap of the two types of metamaterials, the periphery of the scatterer produces obvious circumferential relative displacement with respect to the interior. Similarly, corresponding to the upper boundary of the bandgap, both A2 and B2 vibration mode diagrams indicate the out-of-plane motion at the corner of the substrate foam.

Further, when the filling ratio η_0_ changes (as shown in Figure 5a,b), the start and end frequencies and the RBW of the bandgap exhibit different trends. On the one hand, the lower boundaries of the bandgaps of Type I and II rise slightly with increasing filling ratio and are very close to each other but do not yet reach below 1 Hz. On the other hand, the upper boundary of the bandgaps obviously rises with the increase in the filling ratio, wherein the upper boundary of Type II is not only higher but also able to jump to extremely high levels at high filling ratio. At the same time, the center frequencies of the respective bandgaps of Ref. 1 and Ref. 2 are marked with round black dots in Figure 5c, while the upper and lower boundaries of the bandgaps are indicated by short horizontal lines. Combined with Figure 5d, it is obvious that both Ref. 1 and Ref. 2 just provide a narrower RBW, compared to the Type I and II schemes proposed in this paper.

Therefore, we have learned that the metamaterial structure with hexagonal cross-sectional scatterers has convincing potential to generate a more considerable wide bandgap than those with circular cross-sectional scatterers. Moreover, it cannot be ignored that although Ref. 2 reduces the effective frequency of the bandgap and widens the RBW on the basis of Ref. 1, it still maintains the unit size of 10 m. What is striking is that the Type II scheme proposed in this paper not only reduces the cell feature size by almost an order of magnitude, but also produces a much wider bandgap. In fact, in research and practical applications, these metamaterial units with C6 symmetry proposed in this paper have advantages over the previous configurations (e.g., Ref. 1 and Ref. 2), which possess lower rotational symmetry.

However, it is still critical to note that the lower boundary of the bandgap for Ref. 2 in Figure 5c dips even below 1 Hz, so can that level be reached for the Type II scheme? Naturally, this issue triggers our next work.

### 3.2. Filling Ratio of the Unit and Negative Effective Parameters

After the comparative analysis in the previous section, we have recognized the wide bandgap characteristics of type II metamaterials under large filling ratio. Further, on this basis, this section studies the possibility of this type of metamaterial in generating a CBG at lower frequencies (especially below 1 Hz). More importantly, we interpret the band structure in conjunction with effective parameters with frequency dependence.

**Figure 6:**
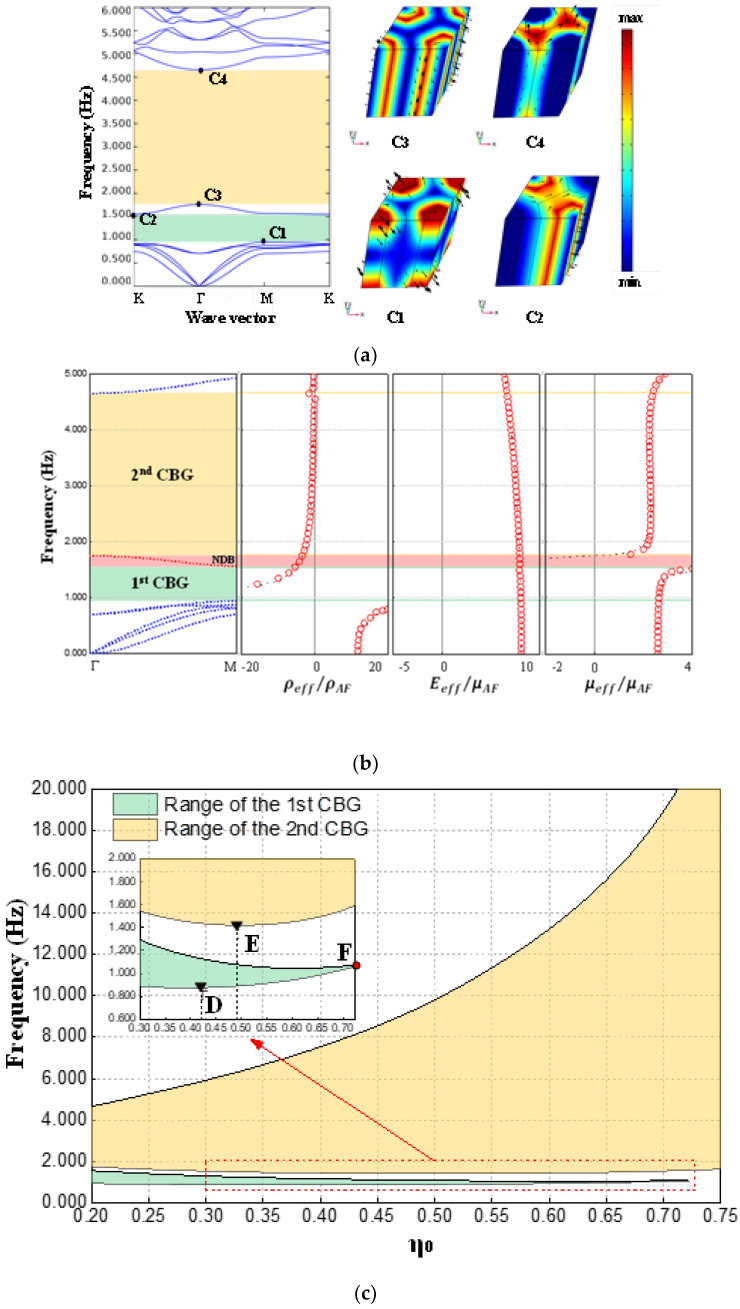

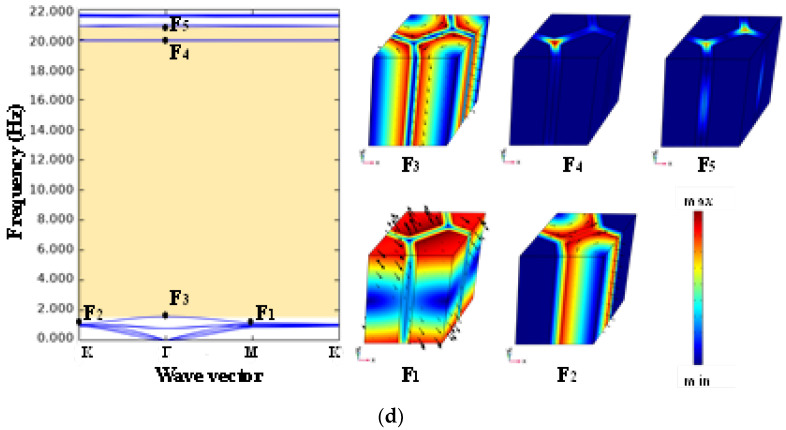
Dispersion relations and concerned vibration mode shapes of Type II EMM when (**a**) η_0_ = 0.200 and H = 2.5*a*_0_, and (**d**) η_0_ = 0.723 and H = 2.5*a*_0_; (**b**) effective material parameters of Type II EMM when η_0_ = 0.200 and H = 2.5*a*_0_; (**c**) range of the CBGs of Type II EMM when the filling ratio varies between 0.200 and 0.750.

According to Figure 4c, it seems that the lower boundary frequency of the CBG can further decrease with the decline in the filling ratio. Then, when the filling ratio is sufficiently lowered to 0.200 and H remains 2.5*a*_0_, the dispersion curves and the bandgap boundary mode diagrams, as shown in Figure 6a, are obtained. Apparently, Figure 6a shows two complete bandgaps, where the FBG lies between 0.946 Hz and 1.551 Hz, while the SBG is situated between 1.759 Hz and 4.657 Hz. For the edge of the SBG, the vibration mode diagrams corresponding to the lower and upper boundary frequencies are shown as C3 and C4, respectively. As the vibration modes corresponding to the bandgap boundaries are almost the same, the SBG in Figure 6a is filled with yellow color as the bandgap in Figure 5b. Actually, we are able to confirm that this kind of bandgap can be classified as the LR bandgap. From the vibration mode diagram C1 corresponding to the lower boundary of the FBG, it can be found that the scatterer has local translation near the surface layer, and the movements near upper and lower layers are reversed. In addition, vibration mode C2 corresponding to the upper boundary of the FBG exhibits a rotational motion mode of the periphery of the scatterer. Unlike the SBG, this FBG is a narrow band-gap dominated by the localized mode of the scatterer alone. To distinguish each other, FBGs of this type are filled with light green.

Further, in order to reveal the origin of the bandgaps, we calculate the effective parameters at this point based on Lai’s method [79] and revised Lai’s method [80]. The results are shown in Figure 6b, where the red circles indicate Lai’s method, while the black dots are the results of the revised version. In this case, all effective parameters are normalized according to the parameters of the substrate material. It is easy to see that the results of the two methods can basically match, but the latter, which has been improved for the long-wave limit case, displays a higher resolution. In the first and second CBGs, $\rho_{eff}<0,E_{eff}>0,\mu_{eff}>0$ and $E_{eff}/\mu_{eff}\geq 1$, so it can be determined that these two bandgaps are dominated by dipole resonance. At the same time, we also find that the FBG and SBG are separated by a negative dispersion band (NDB) with negative curvature (as shown in the red shaded area in Figure 6b). Obviously, within the NDB, the effective parameters are as follows: $\rho_{eff}<0,E_{eff}>0,\mu_{eff}<0$. According to the criterion at Section 2.1, this NDB is unlikely to be utilized for Lamb wave isolation.

In order to investigate the variation laws of the above two kinds of bandgaps more comprehensively, we perform precise calculations and record the frequencies of the bandgap boundaries over a larger range of filling ratios, as summarized in Figure 6c. By observing Figure 5c, it is not difficult to find that the FBG narrows slowly with the increase in the filling ratio η_0_, and its lower boundary reaches the lowest value of 0.876 Hz when η_0_ is about 0.423 (as point D shown in the local enlarged view), while when η_0_ is about 0.723 (as point F), the FBG completely disappears. Here, in order to preliminarily explain the disappearance of the first CBG, the band structure and the bandgap boundary mode diagram at the “vanishing point” F are plotted in Figure 6d. The vibration mode diagrams F1 and F2 show that the characteristic frequency of the local translational mode (represented by point F1) of the surface layer of the scattering body gradually approaches the characteristic frequency of the local rotational mode (represented by point F2) of the outer edge of the scattering body with the increase in the filling ratio. When the characteristic frequencies of the above two vibration modes coincide, the first CBG disappears. In addition, Figure 6c also shows us that the SBG widens rapidly with the increase in the filling ratio, and its lower boundary reaches the lowest value of 1.415 Hz when η_0_ is about 0.490 (as point E shown in the local enlarged view), while its upper boundary can easily break through 20 Hz under the condition of high filling ratios.

### 3.3. The Poisson’s Ratio of the Substrate and the Emerging New Band Gap

**Figure 7:**
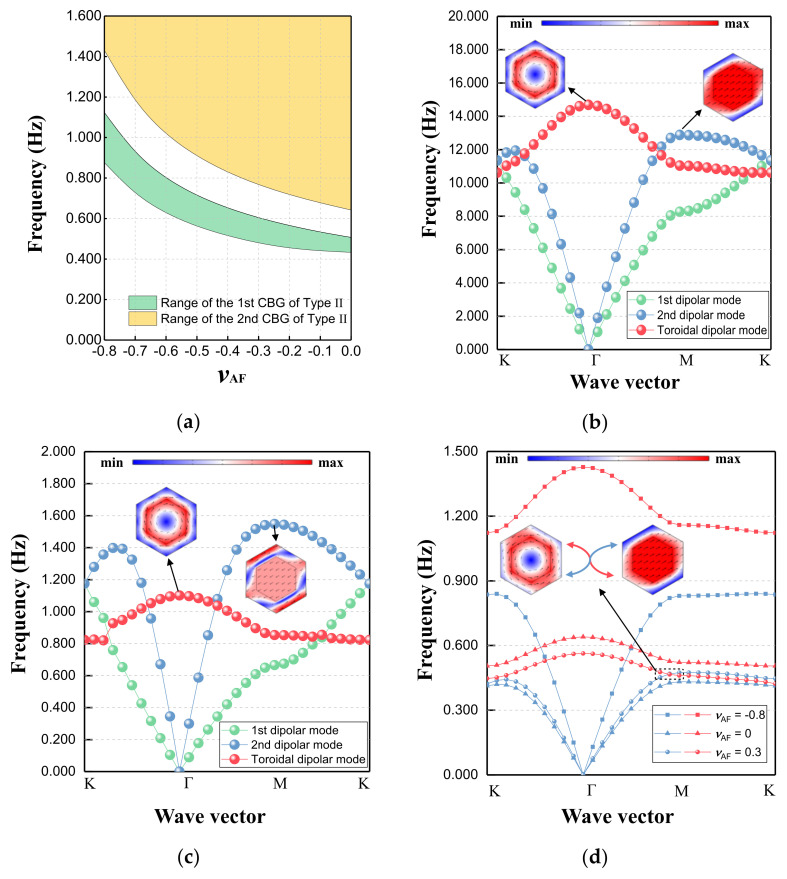
(**a**) Range of the CBGs of Type II EMM when η_0_ = 0.423, H = 2.5*a*_0_, and *ν*_AF_ varies between -0.8 and 0; (**b**) dispersion relations and the representative modes of Type II EMM with η_0_ = 0.423 and H = 2.5*a*_0_ when the substrate is soil and (**c**) rubber; (**d**) dispersion curves of Type II EMM when η_0_ = 0.423, H = 2.5*a*_0_, and *ν*_AF_ varies among 0.3, 0, and -0.8, with the critical state close to the dissolution of degeneracy.

As mentioned in the previous section, the combination of effective parameters determines the formation and location of the CBGs. Thus, when the physical parameters of the substrate auxetic foam, especially the Poisson’s ratio, change, the range of the CBGs will also shift due to the change in the effective parameters. As depicted in Figure 7a, we calculate the boundary frequencies of the FBG and SBG by keeping the filling ratio of point D constant and varying the Poisson’s ratio of auxetic foam (*ν*_AF_) between -0.8 and 0 [48,81]. As the Poisson’s ratio *ν*_AF_ approaches 0, the range of the FBG shrinks and its lower boundary frequency eventually drops to around 0.433 Hz.

In fact, as the substrate, the most distinctive difference between auxetic foam and other conventional materials is the negative Poisson’s ratio property. Of note is the emergence of the new CBG at such a low frequency, which has not been previously reported in auxetic-related metamaterials. Thus, to clarify the formation of this ultralow-frequency bandgap, we conduct an in-depth analysis of the mode evolution of metamaterials with different components.

Specifically, we maintain the filling ratio of the Type II EMM at 0.423 and replace the substrate with soil and rubber for dispersion analysis. The parameters of these two conventional materials are listed in Table 3. For the convenience of observation, the modes independent of the generation of the lowest bandgap can be ignored. In essence, the off-plane mode is not inherently involved in this physical process, so the model can be reduced to the in-plane dimension. As shown in Figure 7b,c, the first three main dispersion curves represent the first dipole mode, second dipole mode, and toroidal dipole mode. For conventional substrate materials, they cannot untangle the entanglement between the basic modes at the bottom. In other words, CBG dominated by dipole resonance cannot be formed here. However, when the substrate becomes the auxetic foam with negative Poisson’s ratio, there is a dissociation between the second dipole mode and the toroidal dipole mode (as shown in Figure 7d). With the increase in negative Poisson’s ratio, the above two modes are further away from each other, and the ultralow-frequency bandgap is then created and widened. In addition, Figure 7d also depicts the degeneracy point when Poisson’s ratio is 0.3, which is extremely close to the critical state where the bandgap is about to be born. Therefore, this ultralow-frequency bandgap can be regarded as an auxeticity-induced version to some extent.

### 3.4. Radius of Circular Hole in the Hollow Scatterer

The previous subsections demonstrate the ability of the Type II EMM to provide a broadband CBG at high filling ratios. Nevertheless, a high filling ratio implies more steel consumption. Therefore, the introduction of the hollow scatterer structure can further save steel usage and, thus, improve the economy of the MMB system.

In this subsection, the Type III EMM, which is formed by hollowing out the scatterer on the basis of the Type II EMM, is investigated. The effect of the hollowing degree of the hollow scattering body (measured by r_1_/*a*_0_) on the bandgap structure of the metamaterial is the focus of this section.

**Figure 8:**
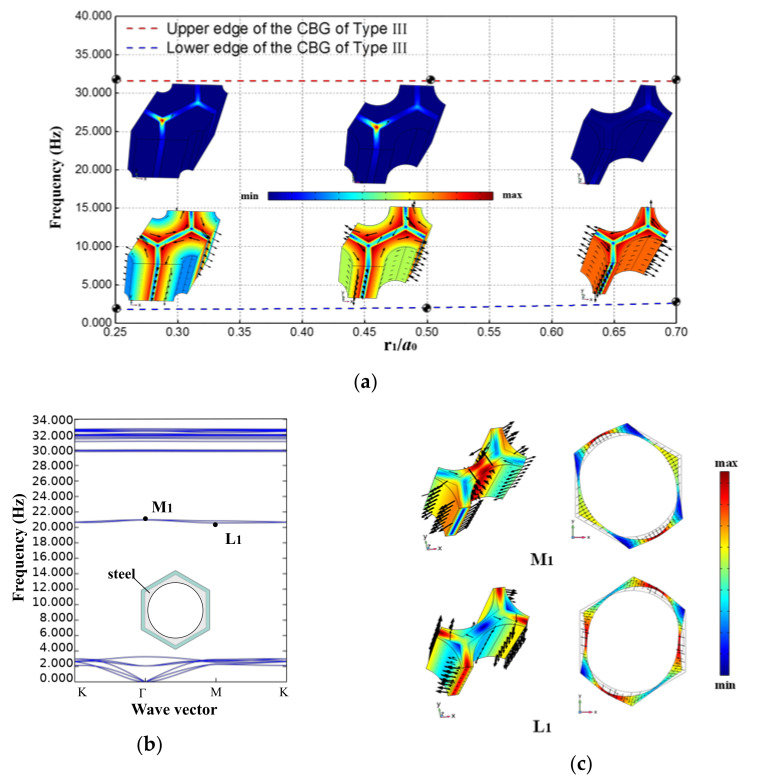
(**a**) Boundaries of the CBG and concerned vibration mode shapes of Type III EMM when b_0_/*a*_0_ = 0.9, H/*a*_0_ = 2.5, and r_1_/*a*_0_ varies between 0.25 and 0.70; (**b**) dispersion relations of Type III EMM when b_0_/*a*_0_ = 0.9, H/*a*_0_ = 2.5, and r_1_/*a*_0_ is 0.75; (**c**) typical vibration mode shapes corresponding to the intervening dispersion curves.

From the trend diagram of the bandgap boundary variation shown in Figure 8a, it can be found that when maintaining b_0_/*a*_0_ = 0.9 and H/*a*_0_ = 2.5, gradually increasing the value of r_1_/*a*_0_ in the range of 0.25-0.70 has almost no effect on the upper boundary (about 31.600 Hz) of the CBG, while it has a weak lifting effect on the lower boundary (from 1.802 Hz to 2.615 Hz). From the typical vibration mode diagram corresponding to the lower boundary of the bandgap, with the increase in r_1_/*a*_0_, the wall of the hollow scatterer becomes thinner, and the frequency of the toroidal dipole resonance increases slightly. Evidently, the locally off-planar resonance of the substrate rather than the scatterer explains the fact that the upper boundary of this CBG remains constant.

However, when the value of r_1_/*a*_0_ is further increased to about 0.75, that is, when the wall of the scattering body becomes quite thin, the CBG will be broken (as shown in Figure 8b). Representatively, the L1 (located at 20.570 Hz) and M1 (located at 20.980 Hz) vibration patterns in Figure 8c indicate that too thin scatterer walls induce unstable resonance in the middle of the boundary, which, in turn, disrupts the continuity of the original forbidden band.

## 4. Isolation of Ultralow-Frequency Lamb Waves by MMB Based on Improved Gradient Design

**Figure 9:**
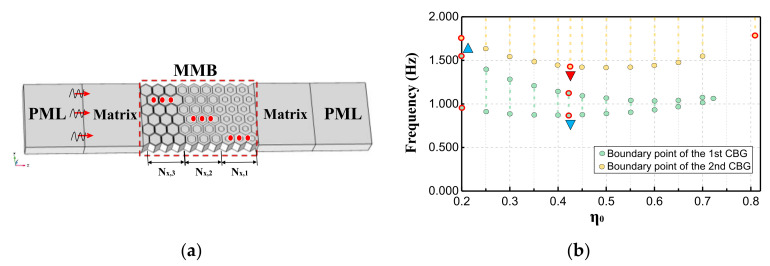
(**a**) Schematic diagram of the MMB system containing single or multiple kinds of EMMs; (**b**) bandgap edge distribution diagram used to provide reference for improved gradient design.

Actually, the periodic structures used for shielding waves in engineering are non-infinite, and in order to verify the accuracy of the bandgap prediction, a finite-scaled system for transmission spectrum analysis is constructed in this paper (Figure 9a). The system mainly consists of perfectly matched layers, soil matrix layers, and an MMB area. Among them, perfectly matched layers (PMLs) are arranged at both ends of the X direction to prevent the wave reflection effect of the boundary. The whole system is matched with the MMB area in the thickness and width direction. In addition, the number of MMB layers along the waveguide direction is denoted by Nx,Q, where the subscript Q represents the EMM layer with diverse parameters. In the tests, the propagation of the Lamb wave is simulated by applying low-amplitude harmonic displacement load (Ain) to the interface between the PML and the left matrix layer. Meanwhile, the output displacement signal (Aout) is collected in the matrix layer at the other end, and the transmission spectrum calculation formula is set as T = 20 × log_10_(Aout/Ain). The MMB is embedded in the middle of the system, which contains one or more types of meta-units, while the top and bottom are established as non-traction boundaries.

It can be seen from Section 3.2 that the lower boundary of FBG and SBG does not change monotonously with the filling ratio, but there is a “bottom point”, which is instructive for using an improved gradient design to overlap the bandgaps. Specifically, ensuring that the upper boundary of the low-frequency bandgap (as shown in the blue triangle in Figure 9b) is higher than the lowest point of the lower boundary of the high-frequency bandgap (as shown in the red inverted triangle in Figure 9b) is the key to reduce the grouping required for gradient design, to design MMB systems with ultra-wideband wave attenuation.

For the purpose of clearly demonstrating the advantages of the gradient MMB system in low-frequency and broadband wave attenuation, several control groups as listed in Table 4 are set-up for the transmission test. Among them, groups 1, 2, and 3 contain only single-component Type II metamaterial units, while group 4 contains all the component units of the first three groups and is arranged in a gradient (as shown in Figure 9a). Moreover, as an optimized design for the fourth group, group 5 replaces partial layers in group 4, replacing Type II EMMs (with η_0_ = 0.81) to Type III (see Table 4 for the specific parameters).

### 4.1. Broadband Suppression of Lamb Waves by MMB

**Figure 10:**
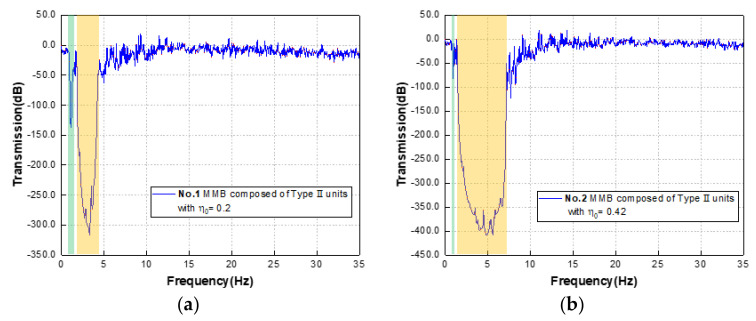

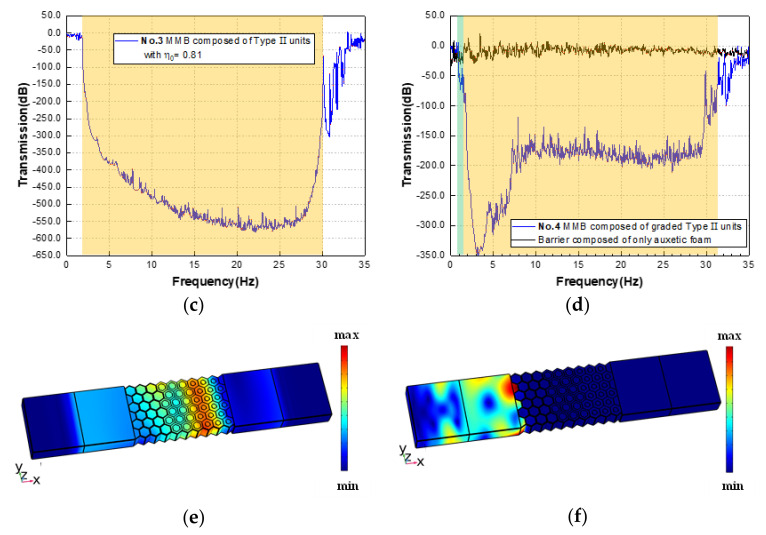
(**a**) Transmission spectrum of group 1, (**b**) group 2, and (**c**) group 3 in the range of 0–35 Hz; (**d**) transmission spectrum of groups 4 and the control group with pure substrate; (**e**) the displacement field of group 4 with frequencies of 0.100 Hz and (**f**) 3.250 Hz.

In the characteristic curves of Figure 10a–c, the transmission test results of groups 1, 2, and 3 are shown, respectively. The green and yellow shaded areas in the figure represent the CBGs predicted by the dispersion curves of the metamaterials of each component. Among them, the predicted bandgaps of group 1 are located at 0.946–1.551 Hz and 1.759–4.657 Hz, the predicted bandgaps of group 2 are at 0.876–1.124 Hz and 1.431–7.929 Hz, and the predicted bandgap of group 3 lies at 1.794–31.613 Hz. By comparing with the significant attenuation region in the transmission characteristic curve, we can find that the accuracy of the prediction range of the bandgap is quite high, and the attenuation effect in the SBG (indicated by the yellow shaded area) is stronger.

Further, the transmission curve (blue solid line) of group 4 is drawn in Figure 10d and the substrate transmission test result (black solid line) without any scatterer is added. From Figure 10d, it can be found that under the effect of the hybrid region of the FBG and SBG, the attenuation of the transmission curve continues, and finally, the broadband attenuation is achieved in the range of 0.876-31.613 Hz. Moreover, compared with the results of the pure substrate transmission test, the attenuation effect of this hybrid MMB system is significant, and the attenuation peak can reach -350 dB. To be more intuitive, the displacement response diagrams (corresponding to group 4) at frequencies outside the bandgap (at 0.100 Hz) and within the bandgap (at 3.250 Hz) are selected, as shown in Figure 10e,f, and then the excellent wave shielding performance of the MMB can be observed. Lamb waves outside the forbidden band can smoothly propagate to the other end (Figure 10e), while waves within the forbidden band are effectively limited to the originating end (Figure 10f).

### 4.2. Broadband Suppression of Lamb Waves by Optimized MMB

**Figure 11:**
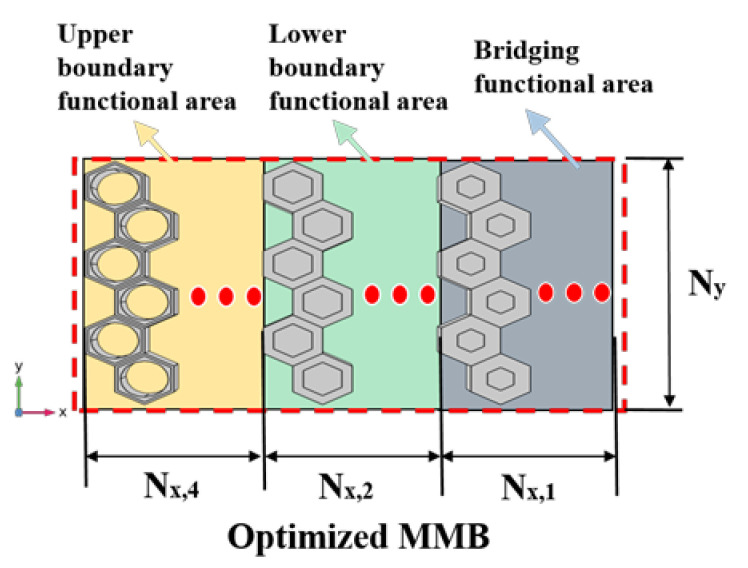
Schematic diagram of the optimized assembled system containing hollowed EMMs.

As it is known from the results of the transmission test of the group 4: the component of η_0_ = 0.81 mainly affects the upper boundary of the attenuation band, the component of η_0_ = 0.42 actually determines the lower boundary of the attenuation band, and the component of η_0_ = 0.20 acts as bridges to ensure the continuity of the attenuation band. Therefore, based on the findings in Section 3.4, we are able to carry out a weight reduction design for the group 4 MMB, the optimized design (group 5) of which is demonstrated in Figure 11. Here, the three functional areas are efficiently integrated to form an assembled MMB. In the comparative tests, the Nx,Q of each area remains as 3, while Ny is 6. Correspondingly, the results of their transmission characteristics are depicted in Figure 11.

**Figure 12:**
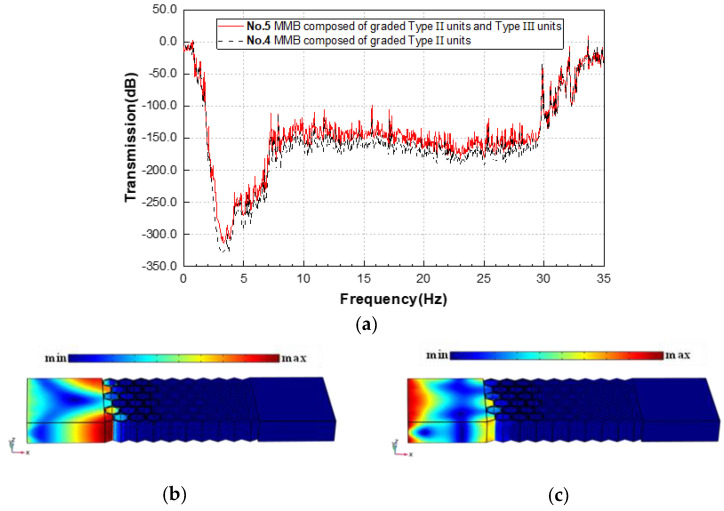

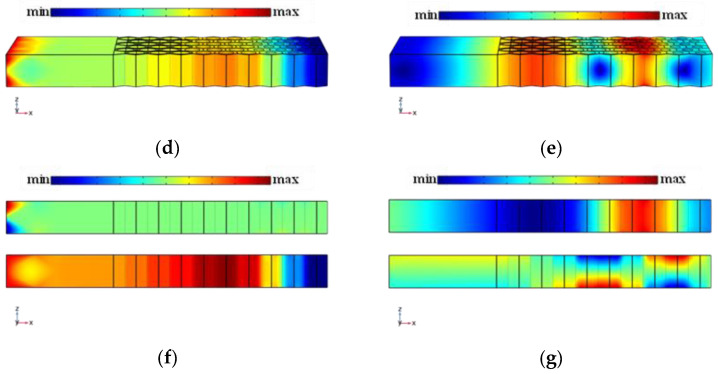
(**a**) Transmission spectrum of MMBs in group 4 and 5 in the range of 0–35 Hz; displacement fields of (**b**) symmetrical mode and (**c**) antisymmetric mode of the 5th group MMB at 3.2 Hz; displacement fields of (**d**) symmetrical mode and (**e**) antisymmetric mode of the 5th group MMB at 0.1 Hz; displacement fields in the Z direction (above) and X direction (below) of (**f**) symmetrical mode and (**g**) antisymmetric mode of the 5th group MMB at 0.1 Hz.

In Figure 12a, the results of group 5 (red solid line) are highly similar to those of group 4 (black dashed line) in the range of the attenuation band. With the RBW up to 1.892 still, this demonstrates the feasibility of optimal design of weight reduction. The introduction of hollow scatterers does not weaken the broadband filtering performance of the original MMB system. More concretely, the strong isolating effect of the improved MMB on symmetric or antisymmetric Lamb waveguides is evident from Figure 12b,c. As a contrast, Lamb wave modes outside the wave shielding region are also reflected in Figure 12d,e, where the symmetric waveguide is predominantly in the form of body modes (as shown in Figure 12f), while the antisymmetric waveguide presents a hybrid of surface and body modes (as shown in Figure 12g).

**Figure 13:**
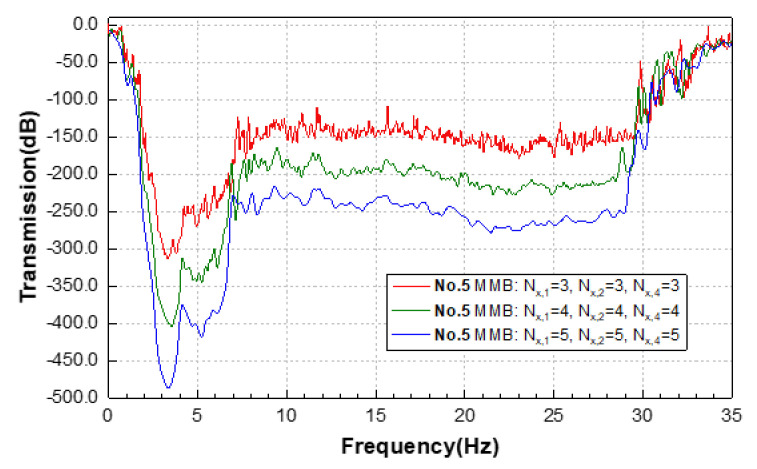
Transmission spectrum of MMBs in group 5 with various layers of component EMMs.

For the setting of an appropriate number of layers of the functional EMMs in the MMB, more comparative tests are performed. More functional layers are set-up in the waveguide direction, in turn, and the detailed transmission test results are shown in Figure 13. The results show that the introduction of more functional layers does enhance the wave attenuation amplitude, especially for the ultralow-frequency region. Normally, each functional EMM occupying about 4 layers is sufficient to provide adequate wave attenuation.

## 5. Conclusions

In this paper, the propagation of Lamb waves in the EMM layer is investigated with reference to effective medium theory and the concept of homogenization under long-wave conditions. Subsequently, two combinations of effective parameters for robust isolation of Lamb waves are summarized and EMMs containing different scatterer configurations are designed accordingly. All types of bandgaps, including the newly discovered ultralow-frequency bandgap, are verified by the effective parameter theory. Finally, a new type of assembled MMB is proposed to isolate Lamb waves based on the improved gradient design, which is further optimized for weight reduction, and its ultralow-frequency and ultra-broadband shielding effectiveness is demonstrated by transmission tests. The conclusions are drawn as follows:

(1) With the auxetic foam as the substrate and the steel core pile as the scatterer, it is feasible to design compact EMMs to isolate Lamb waves at ultralow frequencies such that the effective velocities of both transverse and longitudinal waves are imaginary and the effective modulus of the transverse waves is not greater than the effective modulus of the longitudinal waves.
(2) For the proposed EMMs with a higher symmetry configuration, the negative Poisson’s ratio of the auxetic substrate can be exploited to dissociate the dipole mode and the toroidal dipole mode, thus opening a new complete bandgap at the ultralow-frequency region (below 1 Hz).
(3) The improved gradient design can integrate the ultralow-frequency FBG separated by the NDB with the SBG of wider bandwidth, effectively realizing broadband wave isolation with an RBW of up to 1.892 (or 189.2%), far exceeding those of conventional configurations.
(4) To obtain wider bandwidths, the characteristic size of the required scatterer tends to become larger. In this case, through-holes can be dug inside the scatterer to save steel investment and keep the upper limit of the attenuation band constant. However, there is a limit to the degree of cavitation of the scatterer beyond which the original intact bandgap structure will collapse.

There is no doubt that the superior designability of the auxeticity-induced scheme without introducing additional weight will inspire people to realize efficient wave isolation in the desired frequency domain and further expand the engineering applications of novel assemblable EMMs based on the improving economics and feasibility.

## Figures and Tables

**Table 1:** Possible combinations when the effective wave velocity exists imaginary.

Effective Velocity	Effective Parameters	Properties of Wave Numbers	Wave Velocity
$c_{L}^{2}>0\&c_{T}^{2}<0$	*ρ* > 0, *E* > 0, *μ* < 0	*k_L_*: Im *k_T_*: Im	0 < *c* < *c_L_*
	*ρ* < 0, *E* < 0, *μ* > 0	*k_L_*: Re *k_T_*: Im	0 < *c_L_* < *c*
$c_{L}^{2}<0\&c_{T}^{2}>0$	*ρ* > 0, *E* < 0, *μ* > 0	*k_L_*: Im *k_T_*: Im	0 < *c* < *c_T_*
	*ρ* < 0, *E* > 0, *μ* < 0	*k_L_*: Im *k_T_*: Re	0 < *c_T_* < *c*
$c_{L}^{2}<0\&c_{T}^{2}<0$	*ρ* > 0, *E* < 0, *μ* < 0	*k_L_*: Im *k_T_*: Im	
	*ρ* < 0, *E* > 0, *μ* > 0		

**Table 2:** Geometric parameters of the unit cell (units: m).

*a*_0_	*b*_0_	*r*_0_	*r*_1_	H
2	$\left(0,a_{0}\right)$	$\left(0,\;\frac{\sqrt{3}a_{0}}{2}\right)$	$\left(0,\;\frac{\sqrt{3}b_{0}}{2}\right)$	$2.5a_{0}$

**Table 3:** Materials properties.

Material	Poisson’s Ratio	Young’s Modulus (Pa)	Density (kg/m^3^)
Auxetic foam	-0.80~0	2.50 × 10^4^	120
Rubber	0.47	1.20 × 10^5^	1300
Soil	0.30	2.00 × 10^7^	1800
Steel	0.30	2.10 × 10^11^	7850

**Table 4:** MMB of different components and their serial numbers.

Category	Types of EMMs Included	Parameters of Each Component Layer
No.1	Type II	$\eta_{0}=0.20,N_{x,1}$
No.2	Type II	$\eta_{0}=0.42,N_{x,2}$
No.3	Type II	$\eta_{0}=0.81,N_{x,3}$
No.4	Type II	$\eta_{0}\times N_{x,q}=\left\{0.20\times N_{x,1},0.42\times N_{x,2},0.81\times N_{x,3}\right\}$
No.5	Type II, III	$\mathrm{II}:\eta_{0}\times N_{x,q}=\left\{0.20\times N_{x,1},0.42\times N_{x,2}\right\}$;$\mathrm{III}:b_{0}/a_{0}=0.9\&r_{1}/a_{0}=0.7,N_{x,4}$

## Data Availability

All the data used in this study is already included in the manuscript.
